# The Role of Single-Nucleotide Polymorphisms in the Function of Candidate Tumor Suppressor ALDH1L1

**DOI:** 10.3389/fgene.2019.01013

**Published:** 2019-10-30

**Authors:** Sergey A. Krupenko, David A. Horita

**Affiliations:** ^1^Department of Nutrition, University of North Carolina at Chapel Hill, Chapel Hill, NC, United States; ^2^Nutrition Research Institute, University of North Carolina at Chapel Hill, Chapel Hill, NC, United States

**Keywords:** folate metabolism, ALDH1L1, candidate tumor suppressor, SNPs, human diseases

## Abstract

Folate (vitamin B9) is a common name for a group of coenzymes that function as carriers of chemical moieties called one-carbon groups in numerous biochemical reactions. The combination of these folate-dependent reactions constitutes one-carbon metabolism, the name synonymous to folate metabolism. Folate coenzymes and associated metabolic pathways are vital for cellular homeostasis due to their key roles in nucleic acid biosynthesis, DNA repair, methylation processes, amino acid biogenesis, and energy balance. Folate is an essential nutrient because humans are unable to synthesize this coenzyme and must obtain it from the diet. Insufficient folate intake can ultimately increase risk of certain diseases, most notably neural tube defects. More than 20 enzymes are known to participate in folate metabolism. Single-nucleotide polymorphisms (SNPs) in genes encoding for folate enzymes are associated with altered metabolism, changes in DNA methylation and modified risk for the development of human pathologies including cardiovascular diseases, birth defects, and cancer. ALDH1L1, one of the folate-metabolizing enzymes, serves a regulatory function in folate metabolism restricting the flux of one-carbon groups through biosynthetic processes. Numerous studies have established that *ALDH1L1* is often silenced or strongly down-regulated in cancers. The loss of ALDH1L1 protein positively correlates with the occurrence of malignant tumors and tumor aggressiveness, hence the enzyme is viewed as a candidate tumor suppressor. *ALDH1L1* has much higher frequency of non-synonymous exonic SNPs than most other genes for folate enzymes. Common SNPs at the polymorphic loci rs3796191, rs2886059, rs9282691, rs2276724, rs1127717, and rs4646750 in *ALDH1L1* exons characterize more than 97% of Europeans while additional common variants are found in other ethnic populations. The effects of these SNPs on the enzyme is not clear but studies indicate that some coding and non-coding *ALDH1L1* SNPs are associated with altered risk of certain cancer types and it is also likely that specific haplotypes define the metabolic response to dietary folate. This review discusses the role of ALDH1L1 in folate metabolism and etiology of diseases with the focus on non-synonymous coding *ALDH1L1* SNPs and their effects on the enzyme structure/function, metabolic role and association with cancer.

## Introduction: Folate Metabolism and Cellular Homeostasis

Folate (vitamin B9) is a common name for a group of coenzymes that function as carriers of chemical moieties called one-carbon groups (OCGs) in numerous biochemical reactions. The combination of these folate-dependent reactions constitutes one-carbon metabolism, the name synonymous to folate metabolism. The intracellular folate pool consists of several major coenzyme forms, including tetrahydrofolate (THF) and its derivatives differing by the oxidation state of conjugated OCG (Fox and Stover, 2008; Tibbetts and Appling, 2010). Folate coenzymes and associated metabolic pathways are vital for cellular homeostasis due to their key roles in nucleic acid biosynthesis, DNA repair, methylation processes, amino acid biogenesis, and energy balance (Blom et al., 2006; Fox and Stover, 2008; Tibbetts and Appling, 2010; Locasale, 2013; Fan et al., 2014; Ducker and Rabinowitz, 2017). Folate-dependent biochemical reactions underlying these processes include *de novo* purine and TMP biosynthesis, re-methylation of homocysteine to methionine linked to the production of the universal methyl donor S-adenosylmethionine, degradation of histidine and glycine, interconversion of serine and glycine, and the final step of carbon oxidation to CO_2_ linked with NADPH production (Tibbetts and Appling, 2010; Fan et al., 2014; Baggott and Tamura, 2015; Brosnan et al., 2015). Additional folate-dependent pathways include the clearance of formate (Brosnan et al., 2015) and the formylation of mitochondrial methionyl-tRNA, a process essential for translation initiation in eukaryotic mitochondria (Spencer and Spremulli, 2004; Tucker et al., 2011; Minton et al., 2018). Interestingly, a recent paper reported the direct involvement of one of folate coenzymes, 5,10-methylene-THF, in the methylation of mitochondrial tRNAs with the deficiency of this pathway likely being linked to defective oxidative phosphorylation in human cells (Morscher et al., 2018). This discovery not only extends the list of folate-dependent biochemical reactions and further underscores the indispensable role of the coenzyme but also emphasizes that precise molecular mechanisms underlying folate homeostasis are not completely understood.

Folate is an essential nutrient because humans are unable to synthesize this coenzyme and must obtain it from the diet (Cooper, 1986). Insufficient folate intake ultimately leads to deregulation of cellular homeostasis and is associated with increased risk of certain diseases, most notably neural tube defects (NTDs) (Rock et al., 2000; Fleming, 2001; Mitchell et al., 2004; Moat et al., 2004; Beaudin and Stover, 2007; Strickland et al., 2013; Newman and Maddocks, 2017). For example, periconceptional folate supplementation, in addition to preventing NTDs, has been associated with a significant reduction in the incidence of early spontaneous preterm births (Bukowski et al., 2009). Largely for NTD prevention, the FDA in 1996 approved a mandatory fortification of several types of grain foods in the US with a synthetic form of the vitamin, folic acid (FDA, 1996). The fortification resulted not only in a significant reduction of the incidence of NTDs in the US (Blom et al., 2006), but also improved folate status in the adult population (Jacques et al., 1999).

## Folate Enzymes, Single-Nucleotide Polymorphisms and Diseases

**Figure 1:**
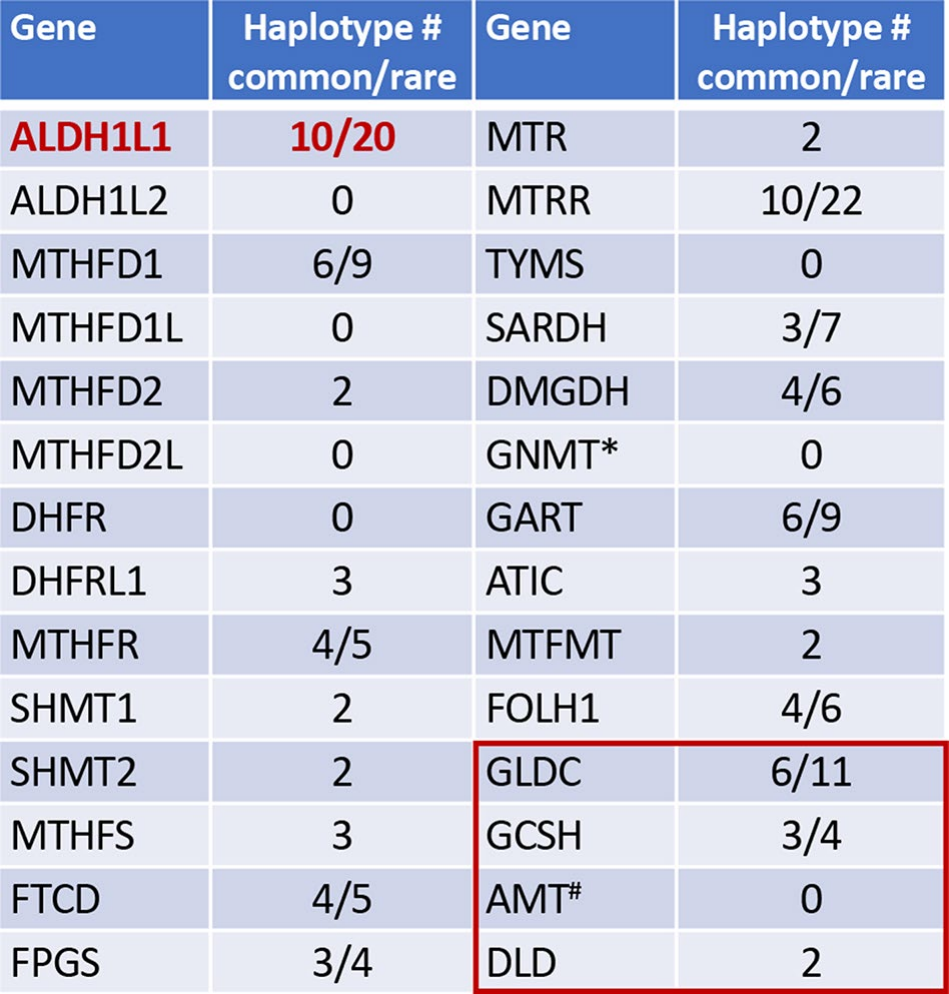
Numbers of common and rare haplotype alleles in genes of folate metabolism (human genome assembly GRCh37/hg19; rare haplotypes have frequency below 1%). *, GNMT is the enzyme regulated by folate. *Red box* indicates four enzymes of the mitochondrial glycine cleavage system, ^#^, the folate dependent enzyme in glycine cleavage. Haplotypes were analyzed using UCSC Genome Browser (https://genome.ucsc.edu).

More than 20 enzymes are known to participate in folate metabolism (**Figure 1**) (Fox and Stover, 2008; Tibbetts and Appling, 2010). They bring OCGs into folate pool, interconvert folate coenzymes, or use OCGs in biosynthetic reactions (Tibbetts and Appling, 2010). Of note, folate enzymes are highly compartmentalized in the cell, being localized to either cytoplasm or mitochondria (Tibbetts and Appling, 2010). Several cytoplasmic folate enzymes can also translocate to the nucleus to enable TMP biosynthesis at specific sites (MacFarlane et al., 2011; Anderson et al., 2012; Field et al., 2014; Field et al., 2015). The nucleus and cytoplasm exchange folate through a simple diffusion, but the mitochondrial membrane is not permeable to folate and shuttling requires a special transporter (Titus and Moran, 2000). Thus, mitochondrial folate metabolism is distinct from cytosolic and uses its own set of enzymes (Tibbetts and Appling, 2010). Several folate reactions in mitochondria parallel those in the cytoplasm; these are catalyzed by homologous enzymes which are products of different genes (Tibbetts and Appling, 2010; Strickland et al., 2011). Folate mitochondrial pathways (i) provide one-carbon groups (in the form of formate) for the cytosolic folate pool, where they are utilized for biosynthetic reactions (Tibbetts and Appling, 2010); (ii) generate NADPH (Fan et al., 2014), or (iii) serve specific mitochondrial functions (Tucker et al., 2011; Morscher et al., 2018; Tani et al., 2018).

Changes in folate metabolism contribute to human pathologies (Stover, 2009), and recent studies underscore the role of several folate enzymes and associated pathways in NTDs and cancer (Jain et al., 2012; Narisawa et al., 2012; Momb et al., 2013; Nilsson et al., 2014; Pai et al., 2015; Piskounova et al., 2015; Ducker et al., 2016; Leung et al., 2017). Alterations in expression or activity of numerous enzymes of folate pathways can either enhance or impair folate metabolism. For example, the increased demand for nucleotides and methylation reactions in cancer cells commonly causes enhanced expression of folate enzymes to maintain the flux of folate-bound OCGs towards biosynthesis, thus supporting increased proliferation (Jain et al., 2012; Ducker and Rabinowitz, 2017; Rosenzweig et al., 2018). Accordingly, several of these enzymes were successfully targeted in cancer chemotherapy (Goldman et al., 2010; Visentin et al., 2012). Further links between the function of folate enzymes and onset of diseases have been clarified in studies using knockout mouse models. Thus, the loss of either MTHFD1L or the folate-dependent glycine cleavage (both localized to mitochondria) causes NTDs in mice (Momb et al., 2013; Pai et al., 2015). Another example is the knockout of folate-regulatory enzyme GNMT: the loss of this protein produces spontaneous tumors in the mouse liver (Martinez-Chantar et al., 2008). It has been also reported that the deficiency in the 10-formyl-THF synthetase activity of cytosolic trifunctional enzyme MTHFD1 is associated with increased incidence of congenital heart defects in mouse embryos (Christensen et al., 2015). Numerous studies also indicate strong gene-nutrient interactions in the folate metabolism regulation. For example, the loss of SHMT1 was insufficient to produce NTDs but caused exencephaly under conditions of maternal folate deficiency (Beaudin et al., 2011; Beaudin et al., 2012).

Single-nucleotide polymorphisms (SNPs) in genes encoding folate enzymes are associated with altered metabolism, changes in DNA methylation and modified risk for the development of human pathologies [reviewed in (Stover, 2011)] including cardiovascular diseases (Klerk et al., 2002), birth defects (Ou et al., 1996; Mills et al., 1999), and cancer (Sharp and Little, 2004; Lightfoot et al., 2010). The most investigated target in these studies was MTHFR (methylene-THF reductase) (Ueland et al., 2001; Hirschhorn et al., 2002; Klerk et al., 2002), which has two common SNPs in the coding region causing non-synonymous amino acid substitutions and creating enzyme variants with reduced activity (Frosst et al., 1995; Weisberg et al., 1998). Numerous SNPs in other key genes of folate pathways, including DHFR (dihydrofolate reductase) (Mishra et al., 2007), MTR (methionine synthase) (Harmon et al., 1999; Ma et al., 1999), TYMS (thymidylate synthase) (Pullarkat et al., 2001), and MTRR (methionine synthase reductase) (Wilson et al., 1999; Gaughan et al., 2001) were linked to human diseases. Of note, the effect of folate pathway gene polymorphisms on disease risk often depends on folate status (Friso et al., 2002; Ulrich et al., 2002; Philip et al., 2015).

## ALDH1L1 Folate Regulatory Enzyme

**Figure 2:**
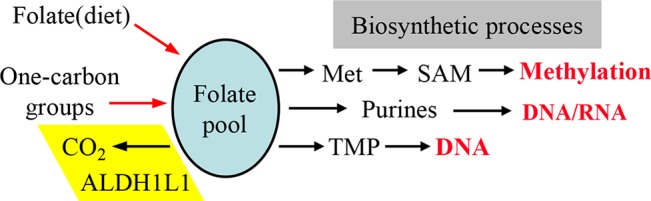
One-carbon groups (derived from amino acid oxidation or formate) enter the folate pool and are directed towards three biosynthetic pathways (methionine, purines and thymidylate synthesis). Note that the enzyme ALDH1L1 diverts these groups from biosynthetic pathways thus serving a catabolic function. Input of folate from diet is required to support the intracellular levels of the coenzyme. SAM, S-adenosylmethionine.

**Figure 3:**
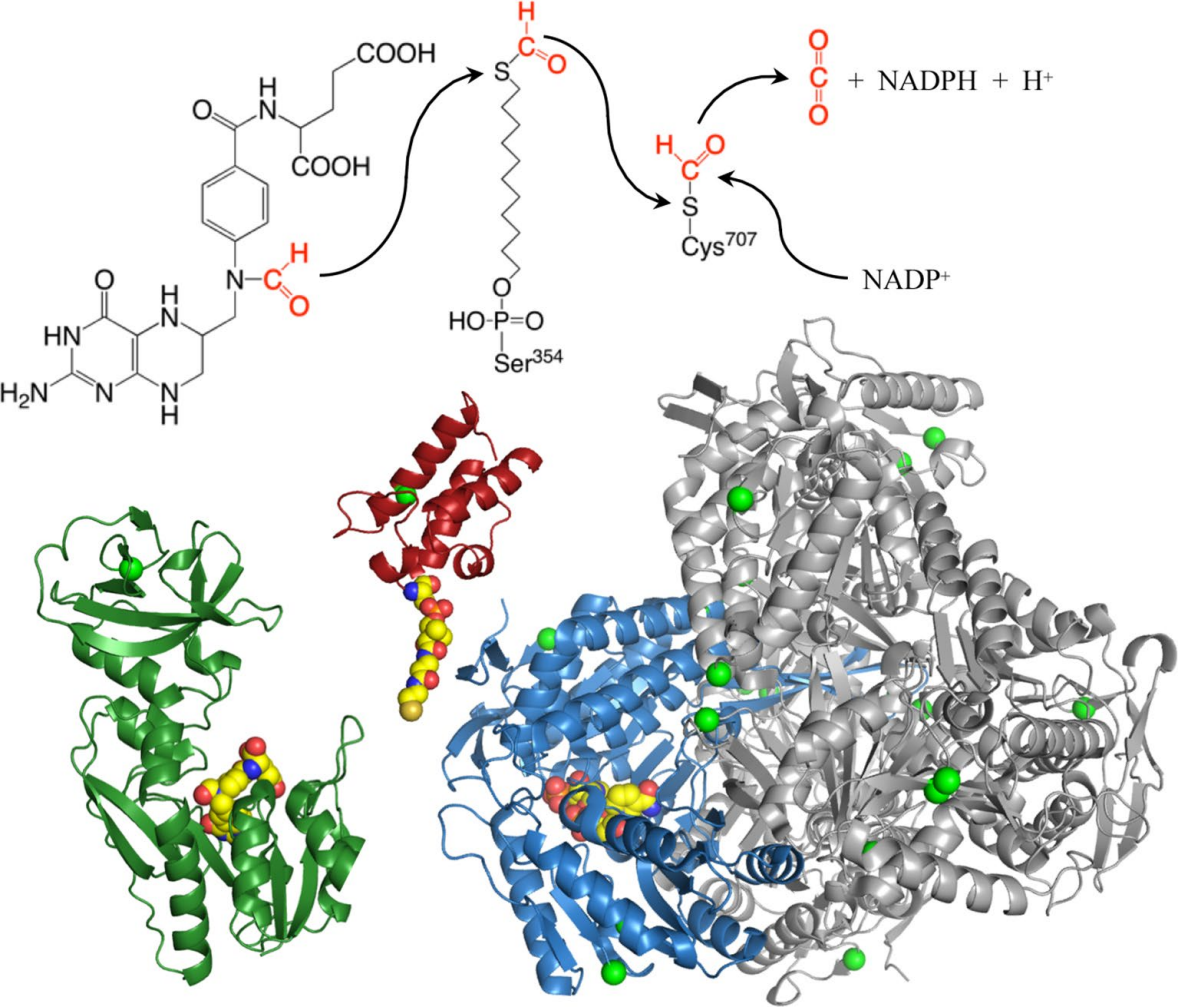
Structures of the N-terminal formyltransferase (*dark green*), central acyl-carrier (*red*, with manually added phosphopantetheinyl moiety), and C-terminal dehydrogenase (*blue* and *gray*) domains of ALDH1L1. *Green spheres* highlight positions of amino acids corresponding to exonic SNPs discussed in the text, other colored spheres show positions of 10-formyltetrahydrofolate (N-terminal domain), phosphopantetheine (intermediate domain), and NADP^+^ (C-terminal domain). Subunit A of the tetrameric dehydrogenase domain is *blue*, subunits B, C, and D are *gray*. The extended phosphopantetheine is critical to the reaction as both the formyl donor (10-formyltetrahydrofolate) and electron acceptor (NADP^+^) are located at the bottoms of clefts in the protein surface. PDB structures are: 4tt8 (N-terminal domain); 2cq8 (intermediate domain); and 2o2q (aldehyde dehydrogenase domain).

ALDH1L1, one of the folate-metabolizing enzymes, converts 10-formyl-THF to THF with simultaneous production of NADPH from NADP^+^ (Krupenko, 2009). By oxidizing the formyl group to CO_2_, this reaction clears the OCG from the cell, thus restricting flux through biosynthetic processes (**Figure 2**). In this way, ALDH1L1 regulates one-carbon metabolism and serves a catabolic function (Krupenko and Oleinik, 2002; Anguera et al., 2006; Krupenko, 2009). ALDH1L1 is active as a tetramer and has a complex structure and catalytic mechanism (**Figure 3**). The *ALDH1L1* gene originated from a natural fusion of three unrelated primordial genes (Strickland et al., 2011; Krupenko et al., 2015), and the resulting protein has a modular organization with three structurally and functionally distinct domains (Krupenko, 2009). The N-terminal folate binding/hydrolase domain structurally resembles methionine-tRNA formyltransferase (Schmitt et al., 1996; Chumanevich et al., 2004) and catalyzes the initial cleavage of the 10-formyl group from 10-formyl-THF (Krupenko et al., 1997a; Chumanevich et al., 2004). The C-terminal dehydrogenase domain forms the tetrameric core and is a structural and functional homolog of aldehyde dehydrogenases (ALDHs) (Krupenko et al., 1997b; Tsybovsky et al., 2007) [hence the assignment of ALDH1L1 to this superfamily of proteins (Marchitti et al., 2008)]. In humans, there are 19 genes encoding for aldehyde dehydrogenases (Marchitti et al., 2008; Koppaka et al., 2012). ALDHs catalyze NAD(P)^+^-dependent irreversible oxidation of a wide variety of endogenous and exogenous aldehydes to corresponding acids, display distinct substrate specificity, and are generally regarded as detoxification enzymes (Marchitti et al., 2008; Koppaka et al., 2012). The ALDH domain of ALDH1L1 shares about 49% of its amino acid sequence with ALDH1, has a typical ALDH fold and by itself catalyzes the oxidation of short-chain aldehydes to corresponding acid using strictly NADP^+^ (Krupenko, 2009). It is not clear whether ALDH1L1 is involved in the utilization of aldehyde substrates *in vivo*. As a part of the ALDH1L1 enzymatic machinery, this domain catalyzes the reduction of NADP^+^ and the oxidation of formyl group to CO_2_ (Krupenko et al., 1997b; Tsybovsky et al., 2007). The two catalytic domains communicate *via* the intermediate domain, which is a structural and functional homolog of acyl carrier proteins (Donato et al., 2007; Strickland et al., 2010). Its prosthetic group, 4′-phosphopantetheine (4′-PP), functions as a flexible arm reaching into the catalytic centers on the N- and C-terminal domains (Horita and Krupenko, 2017) and transporting the reaction intermediate (formyl) from one center to the other (**Figure 3**). The three domains of ALDH1L1 work in concert to enable the conversion of 10-formyl-THF to THF and NADPH production linked to the oxidation of formyl group to CO_2_. Thus, in the case of ALDH1L1 the recruitment of the folate-binding domain extended the substrate specificity of an aldehyde dehydrogenase. Of note, the ALDH family also includes ALDH1L2, the mitochondrial homolog of ALDH1L1 (Krupenko et al., 2010), which is the product of a separate gene [one of the 19 *ALDH* genes (Marchitti et al., 2008)].

That ALDH1L1 serves a regulatory role was determined by several reports that demonstrated the effect of the enzyme on folate and purine pools and on methylation (Champion et al., 1994; Oleinik et al., 2005; Anguera et al., 2006; Oleinik et al., 2006; Hoeferlin et al., 2011). ALDH1L1 is also a key component of the formate degradation pathway, which converts toxic formate to neutral CO_2_, through 10-formyltetrahydrofolate as an intermediate (Strickland et al., 2011). In the cell, formate is directly produced not only from the degradation of 3-methyl-branched fatty acids and the shortening of 2-hydroxy long chain fatty acids (Casteels et al., 2007) but also from the oxidation of methanol present in juices and alcoholic beverages (Hang and Woodams, 2010) and from metabolism of artificial sweetener aspartame (Choudhary and Pretorius, 2017). The first step of the formate degradation pathway, the incorporation of formate into the folate pool, is catalyzed by MTHFD1 and the second rate-limiting step releasing CO_2_ is catalyzed by ALDH1L1 (Neymeyer et al., 1997). It appears that the ALDH1L1-dependent pathway is the only pathway in humans to metabolize formate, and it is more prominent for the clearance of lower, physiological doses of formate (Cook et al., 2001). In further support of this role, decreased expression of ALDH1L1 was observed in cobalamin-deficient rats, likely as a mechanism to divert formate towards methyl group production (MacMillan et al., 2018). ALDH1L1 was also highlighted as a pan-astrocyte marker (Cahoy et al., 2008), but its importance for the astrocyte function is not clear. Interestingly, decreased levels of ALDH1L1 in cerebrospinal fluid were linked to neonatal hydrocephalus in a rat model (Cains et al., 2009). Further studies of this model suggested a role for the enzyme in cerebral folate transport and regulation of folate availability in the brain (Naz et al., 2016; Jimenez et al., 2019). In line with such function, it has been also demonstrated that ALDH1L1 protects folate from degradation in zebrafish embryos, which is a defense mechanism against oxidative stress (Chang et al., 2014; Hsiao et al., 2014). Furthermore, the protective effect of ALDH1L1 on THF degradation has been recently observed in cancer cells (Zheng et al., 2018). These studies provide experimental support for the hypothesis that ALDH1L1 serves as folate depot (Krupenko and Krupenko, 2018).

## Evidence That ALDH1L1 Is a Candidate Tumor Suppressor

ALDH1L1 is most abundant in liver, kidney and pancreas comprising about 1% of total cytosolic protein in hepatocytes (Krupenko, 2009). However, it is not a housekeeping gene and its expression is tissue-specific with some tissues lacking this protein expression (Krupenko and Oleinik, 2002). Furthermore, the enzyme is tightly regulated during mouse brain development (Anthony and Heintz, 2007) and during the progression of NIH3T3 cells through the cell cycle (Khan et al., 2018). In both cases, ALDH1L1 protein is dramatically decreased in proliferating cells but elevated in non-proliferating/resting cells. During mouse brain development, ALDH1L1 expression is likely controlled by transcriptional regulation (Anthony and Heintz, 2007) while in NIH3T3 cells it is rapidly degraded through the ubiquitin-proteasome pathway during the transition from G0/G1 to S-phase (Khan et al., 2018). Because the enzyme limits proliferation by diverting OCGs from biosynthetic to catabolic pathways, its down-regulation could be one of the mechanisms to maintain proliferative state.

In line with its antiproliferative function, ALDH1L1 is often silenced or strongly down-regulated in cancer cell lines and malignant tumors [reviewed in (Krupenko and Krupenko, 2018; Krupenko and Krupenko, 2019)]. This is in strict contrast to other folate enzymes, which are commonly up-regulated in cancer (Jain et al., 2012; Ducker and Rabinowitz, 2017). Several studies have established that the silencing of *ALDH1L1* in human cancers is driven by gene methylation (Oleinik et al., 2011; Dmitriev et al., 2012; Senchenko et al., 2013; Dmitriev et al., 2014; Beniaminov et al., 2018). Methylation takes place in the CpG island, which includes 96 CpG base pairs and covers the promoter, first exon and the part of the first intron in *ALDH1L1* (Oleinik et al., 2011; Beniaminov et al., 2018). Remarkably, a microarray-based global gene expression profiling of approximately 42,000 genes has found that ALDH1L1 was one of the most down-regulated proteins in primary hepatocellular carcinomas and in liver metastases (Tackels-Horne et al., 2001). Analysis of gene expression profiles across 33 human cancer types using The Cancer Genome Atlas (TCGA) data indicated that *ALDH1L1* is more strongly down-regulated in late-stage cancers (Li et al., 2017). Overall, the loss of ALDH1L1 protein positively correlates with the occurrence of malignant tumors and tumor aggressiveness [reviewed in (Krupenko and Krupenko, 2018; Krupenko and Krupenko, 2019)], hence the suggestion that the enzyme is a candidate tumor suppressor (Senchenko et al., 2013).

## SNPs in *ALDH1L1* and Their Association With Pathologies

*ALDH1L1* is located on the minus strand of chromosome 3, spans about 94 thousand nucleotides and may harbor numerous SNPs. Several reports have investigated the functional role of some of these SNPs as well as their associations with diseases. For example, genome-wide association studies (GWAS) revealed that SNPs in *ALDH1L1* are associated with serine to glycine ratio in serum (Dharuri et al., 2013) thus supporting the role of the enzyme as metabolic regulator. Another GWAS analysis identified an association between rs1107366, located about 3800 nucleotides upstream of the *ALDH1L1* transcription start site, and glycine to serine ratios (Xie et al., 2013). This study also indicated that the rs1107366-linked glycine to serine ratio is associated with insulin sensitivity but not with type 2 diabetes. *ALDH1L1* SNPs were also associated with NTDs in Dutch and Chinese Han populations (Franke et al., 2009; Wu et al., 2016).

An interesting study evaluated the effect of two intronic *ALDH1L1* SNPs, rs2276731 and rs2002287, on genome-wide DNA methylation as well as site-specific methylation in normal breast tissues from healthy women (Song et al., 2016). This study identified 57 CpG sites in human genome that were differentially methylated depending on SNPs in six genes of folate metabolism. The strongest association for differential methylation at these sites were with the *ALDH1L1* SNPs. Furthermore, rs2276731 was also associated with a significantly higher global DNA methylation as well as with differential methylation of CpGs within *ALDH1L1* itself. Of note, for both ALDH1L1 SNPs, the pattern of differentially methylated sites was different between whites and blacks (Song et al., 2016). Importantly, a modifying effect on breast cancer incidence of these *ALDH1L1* SNPs has also been reported (Stevens et al., 2007). Here, however, these SNPs have opposite effects: the rs2276731 allele was associated with increased risk whereas the rs2002287 allele was associated with decreased risk of breast cancer.

The rs2276731 SNP could also have a role in the host-gut microbiome interaction. This has been suggested from the 16S rRNA-based analysis of the gut microbiome in 1,126 twin pairs, which thought to calculate the heritability of specific components of the gut microbiota and to find associations between the abundance of specific microbes and host gene alleles (Goodrich et al., 2016). The study identified an association between the host gene *ALDH1L1* (via rs2276731) and the bacteria SHA-98 [unclassified genus of the order SHA-98, phylum Firmicutes (Goodrich et al., 2014)]. It further suggested that this association is linked to the metabolism of formate (as discussed above, ALDH1L1 is a key component of the formate clearance). In addition to the sources listed in the previous section, formate is also a fermentation product which acts as a major interspecies electron carrier promoting syntrophy (Goodrich et al., 2016). Of note, it has been shown that urinary formate excretion significantly correlated with blood pressure (Holmes et al., 2008). Since a SNP in *ALDH1L1* was associated with incident ischemic stroke (Williams et al., 2014), the enzyme might link formate metabolism with the risk of cardiovascular diseases.

**Figure 4:**
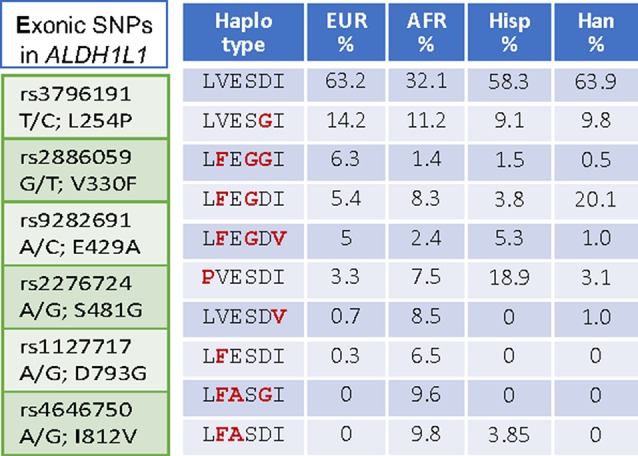
*Left panel*, SNPs in the exonic region causing non-synonymous amino acid substitutions are common in *ALDH1L1*. *Right panel*, SNP-associated haplotypes are markedly different between ethnic populations.

Interestingly, *ALDH1L1* has much higher frequency of non-synonymous exonic SNPs than most other genes for folate enzymes (**Figure 1**). Such SNPs cause amino acid substitutions, could affect the enzyme function, and thus could be relevant to the role of the enzyme in cancer. Curiously, a highly similar mitochondrial homolog, ALDH1L2, which is a product of a separate gene resulted from gene duplication (Krupenko et al., 2010; Strickland et al., 2011; Krupenko et al., 2015), does not have common SNPs (**Figure 1**). SNPs in *ALDH1L1* are common but their effect on metabolism and the etiology of cancer disease is not well understood. Notably, the frequency of exonic SNPs in this gene is highly different between ethnic populations [**Figure 4**; analyzed using UCSC genome browser (Mangan et al., 2014)]. While common SNPs at the polymorphic loci rs3796191, rs2886059, rs9282691, rs2276724, rs1127717 and rs4646750 in *ALDH1L1* exons characterize more than 97% of Europeans, additional common variants are found in African, Hispanic, and Chinese populations (**Figure 4**). Several studies indicated that coding SNPs in *ALDH1L1* are associated with altered risk of certain cancer types. Thus, *ALDH1L1* rs1127717 was associated with the increased risk of hepatocellular carcinoma in Chinese population (1500 cancer patients and 1500 controls were enrolled in this study) (Zhang et al., 2015). Another SNP, rs2276724, could be associated with the post-operative survival of patients with hepatitis B-related hepatocellular carcinoma (Zhu et al., 2017). This study indicates that the effect of the SNP is associated with the expression level of ALDH1L1 mRNA and also depends on the p53 status. An elevated risk of non-Hodgkin lymphoma (NHL) was observed among carriers of the *G* allele at *ALDH1L1 Ex21+31* (p.D793G; rs1127717) (Lee et al., 2007; Lim et al., 2007; Suthandiram et al., 2015). Furthermore, the protective effect of methionine on NHL was associated with *ALDH1L1* SNPs (Lim et al., 2007; Li et al., 2013) suggesting gene-nutrient interactions. Importantly, four exonic SNPs shown in **Figure 4** are associated with leukocyte telomere length (Pusceddu et al., 2017), implicating these polymorphisms in cancer (Sarek et al., 2015; Zhu et al., 2016). Of note, studies investigating *ALDH1L1* SNPs as a risk factor for prostate and renal cancers did not find any associations (Stevens et al., 2008; Gibson et al., 2011), which could suggest the cancer type-specific role of the SNPs. Additionally, the overall effect of *ALDH1L1* SNPs is likely ethnicity-specific (Marini et al., 2016; Wu et al., 2016) and could also be modified by the folate status.

## Potential Impact of *ALDH1L1* Exonic SNPs

The substitution of a single amino acid residue in the protein structure, caused by a SNP, could be mute or could cause significant alterations in protein properties. For example, one of the exonic SNPs in *MTHFR*, C677T, results in the A222V amino acid change in the FAD-binding catalytic domain of the enzyme. This substitution produces a less thermostable protein with reduced catalytic activity (Frosst et al., 1995). Another common exonic SNP in *MTHFR*, A1298C (Weisberg et al., 1998), exists in strong linkage disequilibrium with C677T (Stover, 2011) and results in the E429A enzyme variant. The effect on the enzyme activity of this substitution, which is in the regulatory domain of the protein, is less clear. Initial report indicated that this substitution decreases the enzyme activity though to a lesser extent than the A222V substitution (Weisberg et al., 1998). A later study of purified recombinant human MTHFR concluded that the E429A protein has biochemical properties that are indistinguishable from the wild-type enzyme (Yamada et al., 2001). *In vivo*, however, MTHFR is phosphorylated at multiple residues (Yamada et al., 2005), and both the A222V and E429A mutations are predicted to disrupt phosphorylation of neighboring Ser residues (Shahzad et al., 2013). Notably, the recently solved crystal structure of human MTHFR links the enzyme’s phosphorylation state to its sensitivity to inhibition by S-adenosylmethionine (Froese et al., 2018).

**Figure 5:**
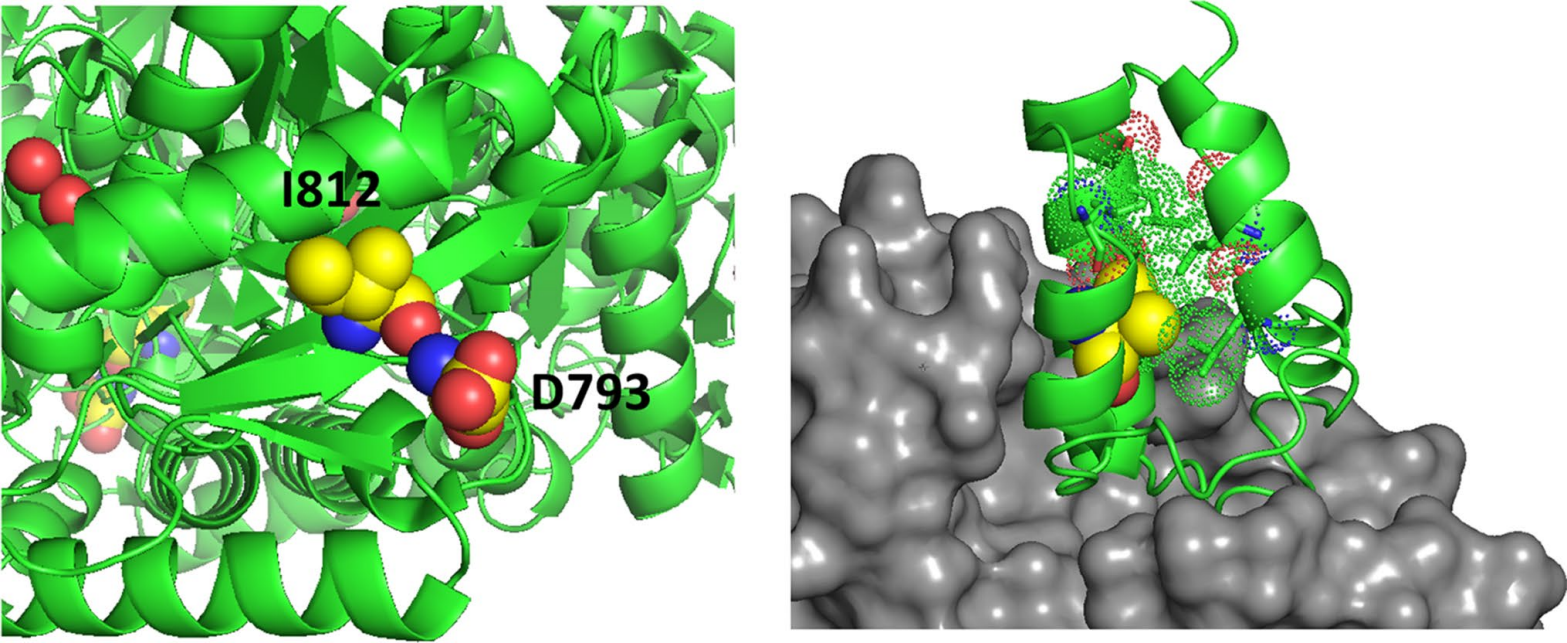
*Left panel*, D793 and I812 are adjacent on parallel β-strands making backbone hydrogen bonds. *Right panel*, the structure of a phosphopantetheinyl transferase (*gray surface*) in complex with an ACP (acyl carrier protein) domain (*green ribbon*) shows that initial modification of the ACP domain serine (*spheres*) requires substantial access to the ACP surface. ACP helices 1 and 2 and the connecting loop lie on the surface of the transferase. The side chain of V330 (*yellow spheres*) packs in the interior of the ACP domain helical bundle. The substitution with Phe (rs2886059) will clash with surrounding residues (*dots*), likely causing a shift of the helix which contacts the transferase domain (*gray surface*) and interfering with binding.

Amino acid substitutions associated with common exonic *ALDH1L1* SNPs are found in each of the functional domains (**Figures 3** and **4**) but their effect on protein properties have not been studied. Analysis of the crystal structures of the ALDH1L1 domains identifies potential important structural roles for residues mutated by these polymorphisms. For example, Ser481 is an α-helix N-cap and its side chain makes a hydrogen bond with Gln549 in a different subunit, suggesting a role in protein oligomerization and stability. Two other residues affected by *ALDH1L1* SNPs, Asp793 and Ile812 (changed to Gly and Val, respectively) are strictly conserved through all species. Interestingly, these residues are adjacent on parallel β-strands and form backbone hydrogen bonds (**Figure 5**). This can be interpreted as a role in supporting protein conformation and stability. Of note, the co-occurrence of both SNPs is not found, suggesting that it perhaps would have too damaging a structural effect if both residues are changed. Our previous studies indicate that point mutations in the ALDH1L1 aldehyde dehydrogenase domain can significantly alter the protein conformation, with some of them impairing the protein’s stability (Tsybovsky et al., 2007; Tsybovsky and Krupenko, 2011; Tsybovsky et al., 2013). Furthermore, a long-range communication between the aldehyde dehydrogenase catalytic center and the NADP^+^-binding domain, observed previously (Tsybovsky and Krupenko, 2011), could transduce the effect of an amino acid substitution to distant domains with an unpredictable effect. In line with this notion, the structure of MTHFR suggests a long-range influence of S-adenosylmethionine binding in the regulatory domain of the enzyme on the catalytic domain some 300 amino acids away (Froese et al., 2018).

The SNP rs2886059 produces the V330F substitution in the intermediate domain of ALDH1L1, close to the modification site where the prosthetic group is attached (**Figure 5**). This substitution introduces a bulky side-chain in the core of the intermediate domain helical bundle which could interfere with the binding of phosphopantetheine transferase (PPTase) (Bunkoczi et al., 2007). PPTase appends the 4′-phosphopantetheinyl moiety to a serine in the intermediate domain and converts inactive apo-ALDH1L1 into active holo-ALDH1L1 (Strickland et al., 2010). Conformational changes associated with other SNPs could interfere with PPTase binding or hinder the ability of the intermediate domain to shuttle reactant between the catalytic domains. The SNP rs3796191 creates the L254P amino acid substitution in the C-terminal lobe of the N-terminal folate binding domain of ALDH1L1. In the structurally homologous enzyme, MTFMT, this sub-domain is responsible for the binding of methionyl-tRNA (Schmitt et al., 1996) but the role of this part of the ALDH1L1 molecule in the enzyme’s function is not clear. It perhaps serves to properly align the folate-binding and the intermediate domains for the acceptance of the formyl group by the 4′-PP arm. Replacement of Leu with Pro will alter and restrict backbone conformation and loop flexibility, and perhaps cause a misalignment between the N-terminal and intermediate domains, impeding access to the folate-binding pocket. In fact, the role of this sub-domain for the proper ALDH1L1 function, likely through the proper orientation of the functional domains, has been demonstrated (Reuland et al., 2006).

Finally, as in the case with MTHFR, coding SNPs can affect ALDH1L1 stability and degradation rate. Towards this end, we have recently demonstrated that ALDH1L1 can be rapidly degraded through the ubiquitin-proteasome pathway (Khan et al., 2018). It is known that protein variants associated with non-synonymous SNPs can be differently degraded by the ubiquitin-proteasome pathway (Siegel et al., 2001; Bandiera et al., 2005). These findings raise the question of whether amino acid substitutions caused by coding SNPs will affect the ALDH1L1 degradation, which would affect the protein function as the proliferation regulator.

## Concluding Remarks

**Figure 6:**
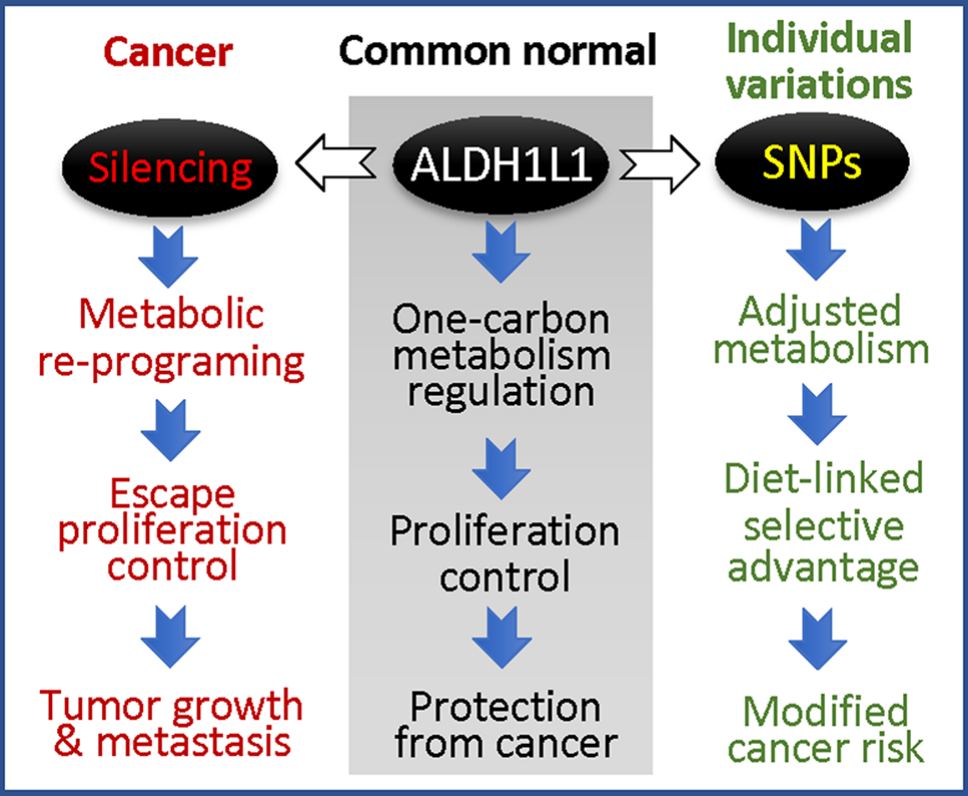
ALDH1L1 is a main regulator of folate metabolism, and its gene is commonly silenced in cancer (the loss of the protein is linked to accelerated proliferation and tumor progression); coding SNPs in this gene are likely to modify cancer risk.

While the phenomenon of ALDH1L1 silencing/down-regulation in cancer is now well recognized (Krupenko and Krupenko, 2018; Krupenko and Krupenko, 2019), the effects of exonic SNPs on the protein function in tumorigenesis and tumor progression are not clear. It is also not known whether this gene is involved in tumor initiation or whether its loss provides selective advantage for tumor progression at later stages. The high prevalence of exonic SNPs causing non-synonymous amino acid substitutions in *ALDH1L1* raises the question of how these SNPs affect cellular metabolism and proliferation regulated by ALDH1L1. If ALDH1L1 polymorphic variants have altered activity or stability/half-life, they are likely to cause the imbalance of intracellular reduced folate pools with a consequent effect on *de novo* purine biosynthesis and amino acid metabolism. Overall, ALDH1L1-dependent metabolic reprogramming associated with functional exonic SNPs could be an important contributor to disease etiology with a more profound effect in populations with certain ALDH1L1 haplotypes (**Figure 6**). With regard to gene-diet interactions, the effect of dietary folate on the ALDH1L1 regulatory role is not clear, and the impact of functional SNPs is yet to be investigated. The understanding of how haplotype-specific effects are modified by folate supplementation could empower precision nutrition approach in disease prevention/treatment. Finally, since ALDH1L1 is involved in formate clearance, it could be an important component of the methanol detoxification pathway (Tephly, 1991). In this regard, it will be interesting to learn whether individuals with different *ALDH1L1* haplotypes have a different susceptibility to methanol toxicity.

## Author Contributions

SK conceived the project, performed analysis of ALDH1L1 gene for coding SNPs and wrote the manuscript. DH performed structural analysis of ALDH1L1 variants and participated in data analysis and manuscript writing.

## Conflict of Interest

The authors declare that the research was conducted in the absence of any commercial or financial relationships that could be construed as a potential conflict of interest.
